# Synthesis of Two Novel 3-Amino-5-[4-chloro-2-phenoxyphenyl]-4*H*-1,2,4-triazoles with Anticonvulsant Activity 

**Published:** 2010

**Authors:** Mohammad Mahdavi, Tahmineh Akbarzadeh, Vahid Sheibani, Maryam Abbasi, Loghman Firoozpour, Sayyed Abbas Tabatabai, Abbas Shafiee, Alireza Foroumadi

**Affiliations:** a *Department of Medicinal Chemistry, Faculty of Pharmacy and Drug Design & Development Research Center, Tehran University of Medical Sciences, Tehran, Iran.*; b * Kerman Neuroscience Research Center, Kerman University of Medical Sciences, Kerman, Iran.*; c * Department of Medicinal Chemistry, Faculty of Pharmacy, Shaheed Beheshti University of Medical Sciences, Tehran, Iran.*; d * Pharmaceutical Sciences Research Center, Tehran University of Medical Sciences, Tehran, Iran.*

**Keywords:** Synthesis, Triazole, Anticonvulsant, Benzodiazepine

## Abstract

Two novel 3-amino-5-(4-choloro-2-phenoxyphenyl)-4*H*-1,2,4-triazole derivatives were prepared and their anticonvulsant activity was measured by evaluation of the ability of these compounds to protect mice against convulsion induced by lethal doses of pentylenetetrazole (PTZ). Diazepam (Sigma) was considered as a positive control drug with anticonvulsant effect [ED_50_ = 1.2 (0.5-1.9) mg/Kg]. Amongst the compounds tested, compound **3**, 3-amino-5- [4-chloro-2-(2-flurophenoxy)phenyl]-4*H*-1,2,4-triazole, showed potent anticonvulsant activity [ED_50_ = 1.4 (1.0-2.2) mg/Kg] compared to diazepam.

## Introduction

Epilepsy is one of the most common neurological disorders, which inflicts more than 60 million people worldwide (1). The main problems associated with antiepileptic drug therapies are uncontrolled seizures and significant toxic side effects. Therefore, there is a continuing demand for new anticonvulsant agents (2). The benzodiazepines are a class of psychoactive drugs with varying anticonvulsant properties, which their effects are mediated by slowing down the central nervous system (3, 4). This class of anticonvulsant compounds has received a lot of attention from the scientific community. Recent studies on structure-activity relationship of benzodiazepines have suggested two features for binding to benzodiazepine receptor: an aromatic ring and a coplanar proton accepting group in a suitable distance. Also, addition of a second out-of-plane, aromatic ring could potentiate binding to the receptor (5). On this basis, preparation and anticonvulsant activity of several derivatives of thiadiazoles , triazoles, and 1,3,4-oxadiazoles have been reported previously (6-9). Moreover, the analgesic and anti-inflammatory activities of various 4-(2-phenoxyphenyl)semicarbazones have been reported (10-11).

**Figure1:**
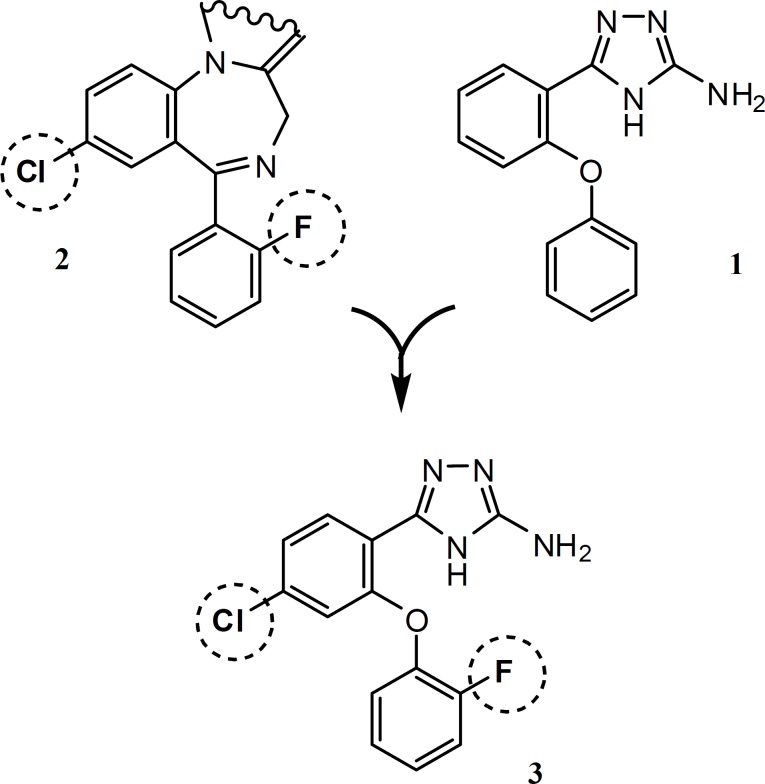
The st ructure of the designed compound**3**, reference molecule **1 **and benzodiazepines **2**.

Among the benzodiazepines, potential anticonvulsant activities of 5-(2-fluorophenyl) benzodiazepines (e.g. Midazolam, Flurazepam and **2**) made them ideal leads for synthesis of new compounds. In the present study, we describe the synthesis of hybrid structure **3**, which is designed by substitution of fluorine and chlorine atoms at the phenyl rings of our previously synthesized compound **1**, in the same positions that are shown on molecule **2 **(Figure 1). Compound **4**, is synthesized to highlight possitive anticonvulsant activity of fluorine substituent in compound **3**.

**Figure 2:**
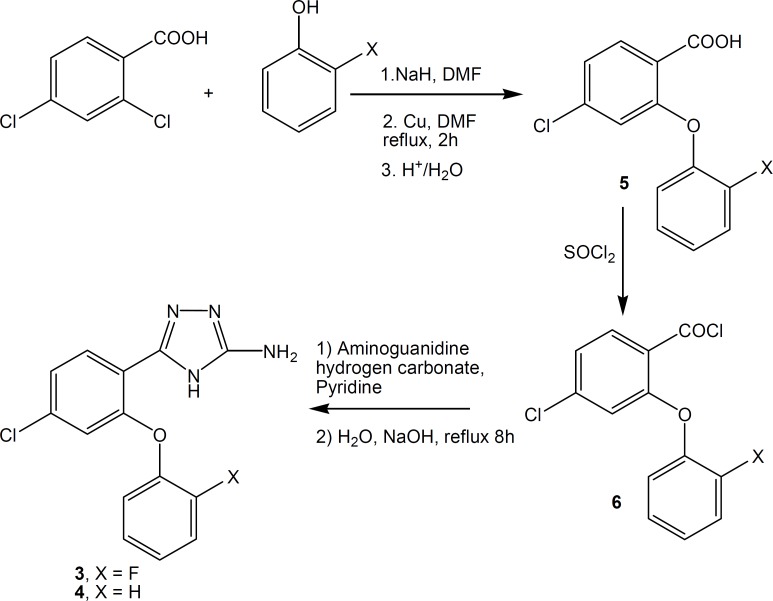
Synthesis scheme of compounds 3 and 4

New compounds were synthesized according to Figure 2. Reaction of 2-phenoxybenzoic acids **5**, with thionyl chloride at 50-55 °C gave corresponding acid chlorides **6**, the intermediates obtained in the preparation of 4H-1,2,4-triazoles (3, 4, 12). Acid chlorides were converted to 4H-3-amino-5-(2-phenoxyphenyl)-1,2,4-triazoles **3 **and **4**, by addition of aminoguanidine hydrogen carbonate followed by cyclization with 5% aqueous solution of sodium hydroxide (5). 

The anticonvulsant activity of synthesized compounds was determined by standard protocol, pentylenetetrazole (PTZ) induced seizure in mice (13). Diazepam (Sigma) was considered as a positive control drug with anticonvulsant effect [ED_50_ = 1.2 (0.5-1.9) mg/Kg]. 

## Experimental

*Preparation of compounds*

The intermediates 4-chloro-2-phenoxybenzoic acids **5**, and 4-chloro-2-phenoxybenzoyl chlorides **6**, were prepared according to the previously described procedures (10, 12).

*3-A*mino-5-[4-chloro-2-(2- flurophenoxy)phenyl]-4H-1,2,4-triazole **3**.

To a stirring solution of aminoguanidine hydrogen carbonate (2.14 g) in dry pyridine (26 mL), at -5°C, a solution of 4-chloro-2-(2-flurophenoxy)benzoic acid chloride (5 g, 17.54 mmol), in dry benzene (26 mL) was added. The stirring was continued for half an hour at -5 °C and then overnight at room temperature. The solvent was evaporated to dryness. To the residue, water (35 mL) was added. The crude white precipitate was suspended in 5% aqueous solution of sodium hydroxide (140 mL) and heated at reflux for 8 h. After cooling the reaction mixture was filtered. The filtrate was acidified with hydrochloric acid and the precipitate was filtered, washed with ethyl acetate and crystallized from ethanol to give 1 g (20%) of **3**, m.p. 282-283 °C. IR (KBr) ν_max_ : 3283, 3227 (NH_2_), 3078 (C-H aromatic). ms: m/z (%) 306 (M^+^+2, 25), 304 (M^+^,100), 249 (32), 152 (32), 95 (32). 1H NMR (CDCl_3_, 500 MHz) δ: 12.09 (brs, 1H, NH), 7.96 (d, 1H, aromatic, *J*=8 Hz), 7.41-7.34 (m, 1H, aromatic), 7.29 (dd, 1H, aromatic, *J*=8 and *J*=1.5 Hz), 7.18 (brs, 2H, aromatic), 7.10-6.77 (m, 2H, aromatic) and 6.02 ppm (brs, 2H, NH_2_).

*3-Amino-5-(4-chloro-2-phenoxyphenyl)-4H-1,2,4-triazole* ***4*** *.*

This compound was prepared from its corresponding precursors as described for the compound **3 **in 28% yield, m.p. 278-280 °C. IR (KBr) ν_max_ : 3270, 3225 (NH_2_), 3085 (C-H aromatic). ms: m/z (%) 288 (M^+^+2, 31), 286 (M^+^,100), 230 (22), 152 (32), 95 (38). 1H NMR (CDCl_3_, 80 MHz) δ: 12.05 (brs, 1H, NH), 7.92 (d, 1H, aromatic, *J*=8 Hz), 7.31-6.80 (m, 7H, aromatic) and 6.00 ppm (brs, 2H, NH_2_).

*Anticonvulsant activity*

Anticonvulsant activity of the synthesized compounds was determined through the evaluation of the ability of the compounds to protect mice against convulsion induced by a lethal dose of PTZ (13).

Male NMRI mice (supplied from Pasteur Institute, Iran) weighting 20–30 g (n=12) were used for pharmacological study. Animals were allowed free access to food and water except during the experiment and housed at controlled room temperature with 12 h light/dark schedule. The animals were transferred to individual cases randomly and allowed to habituate for 30 min before injection of the drug or vehicle.

Test compounds, flumazenil (Sigma) and diazepam (Hoffmann La Roche) were given intraperitoneally as a freshly prepared solution in 50% DMSO and 50% sterile normal saline. The vehicle had no effect on the test system. The test compounds, diazepam and vehicle were administered to groups of 10 mice 30 min before the injection of PTZ (100 mg/kg, ip) and the dead mice were counted 30 min later.

*Statistical analyses*

ED_50_ values and 95% confidence limits were determined using probit-log (dose) model with flumazenil and the test compounds as a categorical covariate and forcing through parallel dose response. Rightward shift of the ED_50_ in logarithmic scale after administration of flumazenil was considered significant if both lower and upper bonds of 95% confidence interval were greater or lower than one. All statistical calculations were performed by SPSS for windows (Rel. 10.0.5.1999. Chicago: SPSS Inc.).

## Results and Discussion

The anticonvulsant activities of tested compounds is reported in Table 1.

**Table 1:** Pharmacological evaluation of synthesized 1,2,4-triazoles

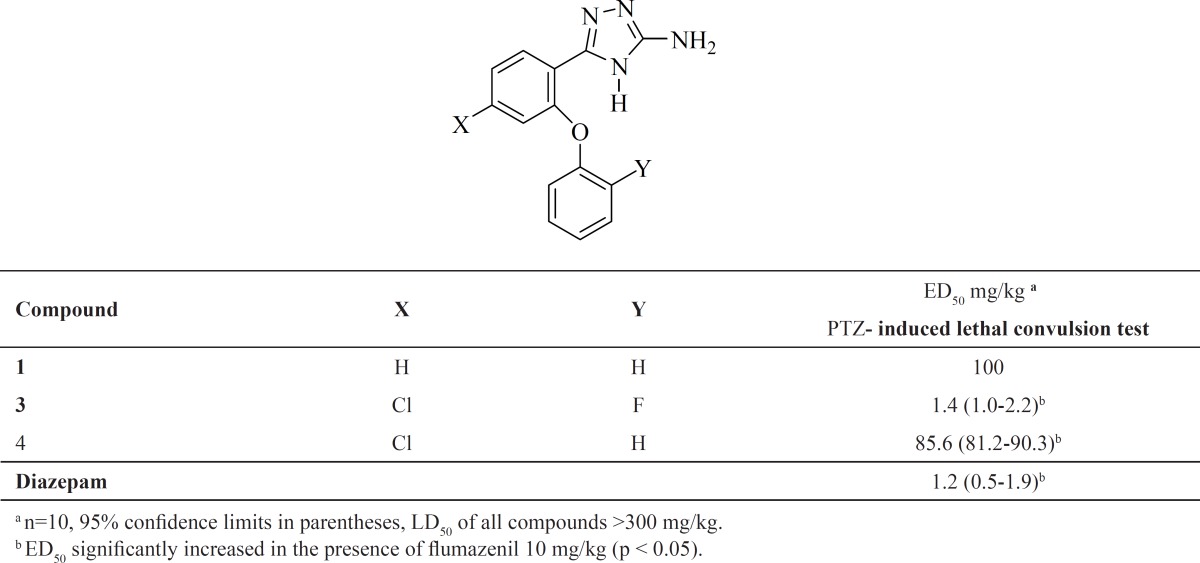

a n=10, 95% confidence limits in parentheses, LD50 of all compounds >300 mg/kg.

b ED50 significantly increased in the presence of flumazenil 10 mg/kg (p < 0.05).

 The anticonvulsant activity of compounds **3 **and **4**, showed that compound **3**, having similar substitutions to the reference molecule **2**, has potent anticonvulsant activity [ED_50_ = 1.4 (1.0-2.2) mg/Kg] compared to diazepam [ED_50_ = 1.2 (0.5-1.9) mg/Kg]. Replacement of the fluorine atom with hydrogen (compound **4**), significantly reduced the anticonvulsant activity [ED_50_ = 85.6 (81.2-90.3) mg/Kg]. However, compound **4**, was more active than corresponding unsubstituted compound **1 **[ED_50_ = 100 mg/Kg]. 

For evaluation of benzodiazepine receptors involvement in the anticonvulsant activity of the test compounds, flumazenil (10 mg/kg, i.p.) as a benzodiazepine receptor antagonist was used 10 min before injection of the compounds. ED_50_ was significantly increased in the presence of flumazenil 10 mg/kg. The fact that the activity of compounds **3 **was significantly reduced by flumazenil, a benzodiazepine antagonist, confirms that this effect is mediated through benzodiazepine receptors.
